# Vitamin E δ-tocotrienol triggers endoplasmic reticulum stress-mediated apoptosis in human melanoma cells

**DOI:** 10.1038/srep30502

**Published:** 2016-07-27

**Authors:** Marina Montagnani Marelli, Monica Marzagalli, Roberta M. Moretti, Giangiacomo Beretta, Lavinia Casati, Raffaella Comitato, Giovanni L. Gravina, Claudio Festuccia, Patrizia Limonta

**Affiliations:** 1 Department of Pharmacological and Biomolecular Sciences, Università degli Studi di Milano, Milano, 20133, Italy; 2 Department of Pharmaceutical Sciences, Università degli Studi di Milano, Milano, 20133, Italy; 3 Department of Medical Biotechnologies and Translational Medicine, Università degli Studi di Milano, Milano, 20129, Italy; 4 Council for Agricultural Research and Economics, Food and Nutrition Research Centre, Roma, 00178, Italy; 5 Department of Applied and Biotechnological Clinical Sciences, Università degli Studi dell’Aquila, L’Aquila, 67100, Italy

## Abstract

Malignant melanoma is the leading cause of death from skin cancer. Drug toxicity and resistance represent a serious challange for melanoma treatments. Evidence demonstrates that natural compounds may play a crucial role in cancer prevention, growth and progression. Vitamin E tocotrienols (TT) were shown to possess antitumor activity. Here, we analyzed the effects of δ-TT on melanoma cell growth and the involvement of the endoplasmic reticulum (ER) stress in this activity. The experiments were performed on human melanoma cell lines, BLM and A375. δ-TT exerted a significant proapoptotic effect on both cell lines, involving the intrinsic apoptosis pathway; importantly, this compound did not affect the viability of normal human melanocytes. In melanoma cells, δ-TT exerted its antitumor effect through activation of the PERK/p-eIF2α/ATF4/CHOP, IRE1α and caspase-4 ER stress-related branches. Salubrinal, an inhibitor of the ER stress, counteracted the cytotoxic activity of δ-TT. *In vivo* experiments performed in nude mice bearing A375 xenografts evidenced that δ-TT reduces tumor volume and tumor mass; importantly, tumor progression was significantly delayed by δ-TT treatment. In conclusion, δ-TT exerts a proapoptotic activity on melanoma cells, through activation of the ER stress-related pathways. δ-TT might represent an effective option for novel chemopreventive/therapeutic strategies for melanoma.

Malignant melanoma is the deadliest skin cancer; its incidence has been increasing faster than any other cancer, with a 2.6% annual increase over the last decade^1^. The majority of melanomas are diagnosed in the early stage and are curable with surgical resection; however, the prognosis of late stage melanomas is still poor. Alkylating agents (dacarbazine and temozolomide) and cytokines (interferon-α and interleukin-2) represent the first treatment options; however, resistance easily develops with serious side effects^2^. Targeted therapy was introduced in melanoma treatment. The V600E mutation (valine at codon 600 is substituted by glutamic acid) of the *BRAF* oncogene is present in approximately 50% of patients, leading to the activation of the mitogen-activated protein kinase (MAPK) pathway; on the other hand, about 30% of melanomas harbour the *NRAS* mutation, known to be associated with increased activation of both the MAPK and the phosphoinositide 3-kinase (PI3K)/Akt pathways^3^. Molecular targeted therapy consists of *BRAF* inhibitors, such as vemurafenib and dabrafenib, or MEK inhibitors, such as trametinib. These compounds were initially associated with positive clinical results; however, a rapid development of resistance was found to occur in most patients^4^. Immune checkpoint inhibitors were developed for the treatment of aggressive melanomas. Ipilimumab, a monoclonal antibody against the CTLA-4 T lymphocyte receptor, and nivolumab and pembrolizumab, monoclonal antibodies against the inhibitory programmed cell death-1 (PD-1) receptor expressed on activated T cells, were approved by the US Food and Drug Administration (FDA)^4^. However, these compounds did not provide the expected improvement on overall survival, being accompanied by severe toxicity^5^. Based on these disappointing observations, combination treatments targeting different intracellular pathways are currently investigated as potential effective therapeutic strategies for aggressive melanomas^6^.

The role of natural dietary components in cancer growth and progression has become a very popular subject. About 36% of the small molecule compounds approved by FDA between 1999 and 2008 are natural products or their derivatives^7^. Moreover, the role of dietary factors in preventing cancers was investigated in a large body of epidemiological studies. Natural compounds, such as epigallocatechin-3-gallate (EGCG), resveratrol, lycopene, polyunsaturated omega-3 fatty acids (PUFA) and genistein, were reported to exert antitumor effects on several cancer cell lines^8^,^9^. These compounds were also shown to possess chemopreventive activity and to potentiate the antitumor effects of standard treatments^10^,^11^.

Vitamin E is a family composed of α, β, δ and γ-tocopherols and the corresponding four tocotrienols (TTs). TTs, in particular, were widely shown to exert health-promoting effects in different chronic diseases, based on their powerful neuroprotective, antiinflammatory, antioxidant and cholesterol lowering potentials^12^,^13^. Evidence has also accumulated demonstrating the more potent anticancer effects of TTs (δ and γ-TT in particular) compared with tocopherols in tumors^14^,^15^. The mechanisms of the antiproliferative properties of tocotrienols are still under investigation and they seem to involve different intracellular pathways^16^,^17^,^18^,^19^.

The endoplasmic reticulum (ER) stress response is a cellular process that can be triggered by different conditions that cause imbalance in intracellular homeostasis. ER stress, which severely impairs protein folding, can be induced by different physiological and pathological conditions^20^, as well as by a number of compounds of synthetic or natural origins^21^,^22^. Cells react to ER stress with an initial defensive process, the so called unfolded protein response (UPR), aimed at restoring homeostasis by enhancing protein folding capacity^23^; however, in conditions of severe stress, misfolded proteins accumulate in the ER ultimately triggering a set of prodeath programs (the *yin* and *yang* principle)^21^. Three major proteins are known to act as stress sensors in the ER: double-stranded RNA-dependent protein kinase PKR-like ER kinase (PERK), inositol-requiring enzyme 1α (IRE1α), and activating transcription factor 6 (ATF6)^24^. In physiological conditions, these sensors are inactivated by the association with the chaperon protein immunoglobulin-heavy-chain-binding protein (BiP, also known as GRP78). Under severe ER stress, BiP dissociates from the sensors triggering their activation; each of these proteins is coupled with a cytosolic pathway and each pathway converges to apoptosis^25^,^26^,^27^. In particular, transcription factor ATF4, activated by the PERK/eIF2α pathway, stimulates the expression of the proapoptotic protein CHOP (C/EBP homologous protein, also called GADD153). IRE1α catalizes an unconventional splicing of the X box binding protein 1 (XBP1). However, under severe ER stress, IRE1α leads to downstream activation of the JNK and p38 MAPK pathways, leading to the activation of apoptosis-related proteins, such as CHOP and caspase-4^26^,^27^,^28^. It is recognized that CHOP is a major player in mediating the ER stress-induced apoptosis pathways.

ER stress is implicated in the pathogenesis of a variety of diseases (neurodegeneration, inflammation, cancer)^20^,^29^,^30^,^31^. Emerging evidence indicates that pharmacological targeting of ER stress can represent an effective therapeutic strategy to treat tumors^21^,^27^,^28^. Different natural compounds have been shown to induce ER stress-mediated death in cancer cells^32^.

Here, we investigated the effects of δ-TT on melanoma cell growth, both *in vitro* and *in vivo*, and the involvement of the ER stress signaling in its activity.

## Results

### δ-TT decreases cell viability and exerts a cytotoxic effect on melanoma cells

**Figure 1:**
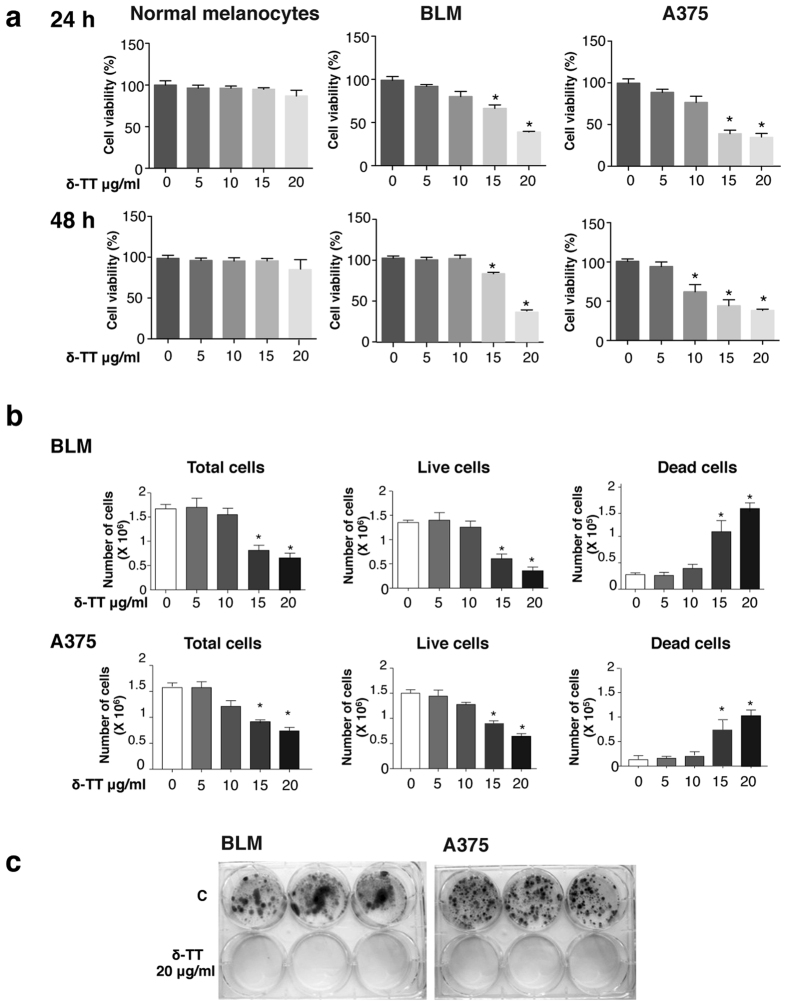
δ-TT decreases cell viability and exerts a cytotoxic effect on BLM and A375 melanoma cells, without affecting the growth of normal human melanocytes. (**a**) Melanoma cells and normal human melanocytes were treated with δ-TT (5–20 μg/ml) for 24 or 48 h. Cell viability was then evaluated by MTT assay. (**b**) Melanoma cells were treated with δ-TT (5–20 μg/ml) for 24 h. Total, live and dead cells were evaluated by Trypan blue exclusion assay. (**c**) Melanoma cells were treated with δ-TT (20 μg/ml) for 72 h and then, after withdrawal of the treatment, were left to grow for 7–10 days, dependently on the cell line-specific proliferation rate. A colony-formation assay was performed to evaluate the ability of the cells to proliferate forming colonies (dimensions of the colonies) and the survival of colony-forming cells (number of colonies). Each experiment was repeated at least three times. Data in (**a,b**) represent mean values ± SEM and were analyzed by Bonferroni’s test after one-way analysis of variance. *p < 0.05 vs 0, controls.

BLM and A375 cells, or normal human melanocytes, were treated with δ-TT (5–20 μg/ml) for 24 h or 48 h; cell viability was assessed by MTT assay. Figure 1a shows that the tocotrienol dose-dependently inhibited the number of viable melanoma cells at both time intervals, being significantly effective at 10–20 μg/ml (Fig. 1a). These doses correspond to those previously reported for the antitumor activity of tocotrienols in cancer cell lines^33^,^34^,^35^. Strikingly, the same treatment schedule did not affect the growth of human normal melanocytes at both time intervals (Fig. 1a).

To investigate whether δ-TT might induce melanoma cell death, BLM and A375 cells were treated with different doses of δ-TT (5–20 μg/ml) for 24 h, then floating (dying) and adherent (living) cells were harvested, stained with 0.4% of Trypan blue (1:1) and counted with an automated cell counter, differentiating from total, living and dead cells. Figure 1b shows that, in both BLM and A375 cells, δ-TT induced a dose-response effect with a significant reduction of total and viable cells, and a parallel significant increase of dead cells, stained with Trypan blue (Fig. 1b).

To confirm the cytotoxic effect of δ-TT on melanoma cells, a colony formation assay was performed. BLM and A375 cells were treated with δ-TT (20 μg/ml) for 72 h and then, after withdrawal of the treatment, cells were left to grow for 7–10 days, dependently on the cell line-specific proliferation rate (36 h for BLM cells and 24 h for A375 cells), to assess: 1) the ability of the cells to proliferate forming colonies (dimensions of colonies); and 2) the survival of colony-forming cells (number of colonies). Crystal violet staining evidenced that control cells were able to grow and proliferate to form colonies. On the other hand, none of the treated cells survived upon δ-TT treatment and colony formation was completely prevented, indicating a cytotoxic, rather than a cytostatic, effect of this compound on melanoma cells (Fig. 1c).

### δ-TT affects the expression of apoptosis-related proteins in melanoma cells

**Figure 2:**
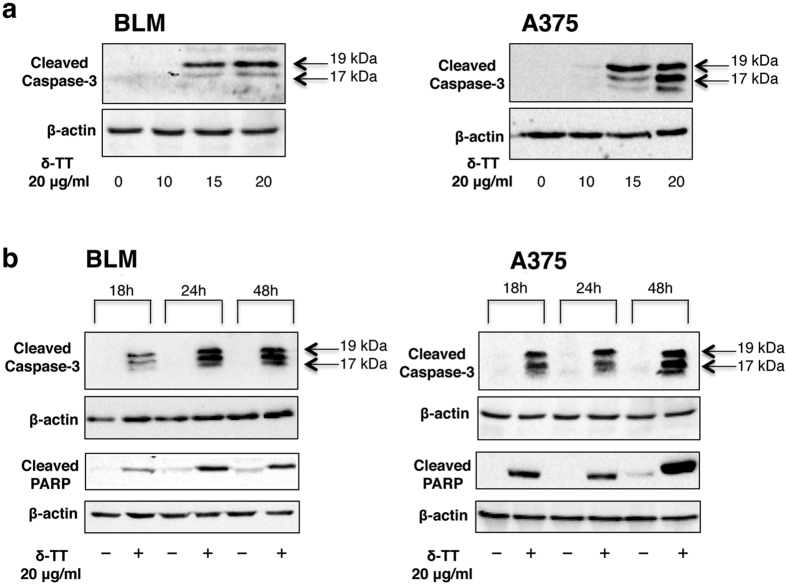
δ-TT affects the expression of apoptosis-related proteins in BLM and A375 melanoma cells. (**a**) Melanoma cells were treated with δ-TT (10–20 μg/ml) for 48 h. Western blot analysis was carried out with anti-caspase-3 (1:500) antibody. (**b**) Melanoma cells were treated with δ-TT (20 μg/ml) for 18–48 h. Western blot analysis was carried out with anti-caspase-3 (1:500) and anti-PARP (1:1,000) antibodies. β-actin expression was evaluated as a loading control. 0, controls. One representative of three different experiments, for each of the analyses performed, is shown.

Apoptosis is highly dependent on the activation (cleavage) of caspases, such as the effector caspase-3, and their direct downstream targets, such as PARP (Poly (ADP-ribose) polymerase), involved in DNA repair. Activated caspase-3 cleaves this enzyme, preventing DNA damages repair and contributing to cell death. To verify whether δ-TT might induce apoptosis, cells were treated with different doses (10–20 μg/ml) of δ-TT for 48 h or with 20 μg/ml for 18–48 h. By Western blotting we could observe that the levels of cleaved caspase-3 increased after δ-TT treatment at the doses of 15 and 20 μg/ml in both BLM and A375 cells (Fig. 2a). A time-course increase of cleaved caspase-3 levels was also observed at the dose of 20 μg/ml at all time intervals in both cell lines (Fig. 2b).

Western blot analyses were then performed to assess the activation of the apoptosis process, by verifying the cleavage of PARP. Accordingly to the time-course cleavage of caspase-3, cleavage of PARP occurred after the treatment of BLM and A375 cells with δ-TT (20 μg/ml) for 18, 24 or 48 h (Fig. 2b).

### δ-TT affects the expression/intracellular localization of proteins involved in the intrinsic apoptosis pathway in melanoma cells

**Figure 3:**
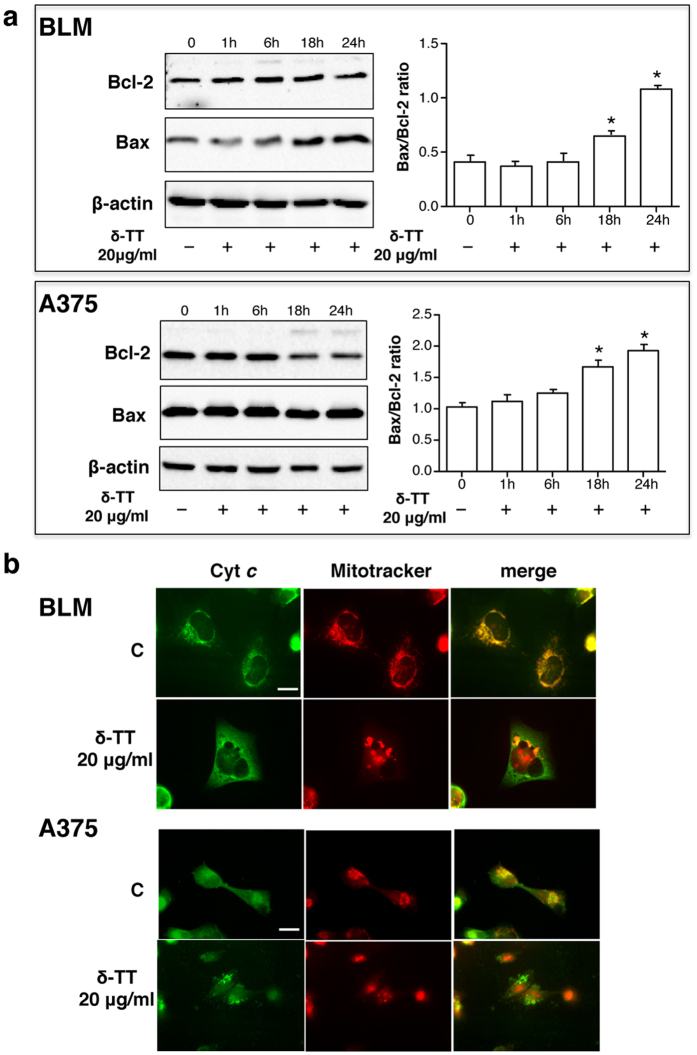
δ-TT affects the expression/intracellular localization of proteins involved in the intrinsic apoptosis pathway in BLM and A375 melanoma cells. (**a**) Melanoma cells were treated with δ-TT (20 μg/ml) for 1–24 h. Western blot analysis was carried out with anti-Bcl-2 (1:1,000) and anti-Bax (1:500) antibodies. β-actin expression was evaluated as a loading control. One representative of three different experiments, for each of the analyses performed, is shown. A densitometric analysis of the bands obtained was performed and the relative Bax/Bcl-2 ratios in both cell lines were calculated. *p < 0.05 vs 0, controls (**b**) Melanoma cells were treated with δ-TT (20 μg/ml) for 24 h and the intracellular localization of cytochrome *c* was evaluated by immunofluorescence analysis. Specifically, cells were incubated for 30 min with Mitotracker (250 nM), fixed and stained with cytochrome *c* antibody (1:50) followed by the FITC-conjugated secondary antibody and DAPI. Cyt *c*, cytochrome *c*. C, controls. One representative of three different experiments performed is shown. Scale bar, 5 μm.

Proteins, such as Bax and Bcl-2, are involved in the intrinsic apoptosis pathway. An increased ratio between Bax (proapoptotic) and Bcl-2 (antiapoptotic) leads to the disruption of the mitochondrial outer membrane potential (MOMP) and to the release of cytochrome *c*. Figure 3a shows that, in BLM cells, an increased expression of Bax occurred after 18 and 24 h of treatment with δ-TT (20 μg/ml); Bcl-2 expression was unaffected by the treatment. In A375 cells, δ-TT induced a reduction in Bcl-2 expression at 18–24 h; no changes in the expression levels of Bax were observed (Fig. 3a). A densitometric analysis has been performed on the protein bands shown in this Figure and the Bax/Bcl-2 ratios have been evaluated. The results obtained indicate that, in both cell lines, an increased Bax/Bcl-2 ratio occurs at 18 and 24 h of treatment, indicative of alterations in MOMP (Fig. 3a). In line with these observations, by immunofluorescence analysis, we found that δ-TT treatment (24 h) induced a different localization of cytochrome *c*, that was diffused in the cytosol and did not overlap with mitochondria (as evaluated by Mitotracker staining) (Fig. 3b), demonstrating that δ-TT induces cytochrome *c* release in both cell lines.

### δ-TT increases the expression of ER stress-related proteins in melanoma cells

Experiments were performed to investigate whether δ-TT might induce ER stress-mediated apoptosis in melanoma cells. As a positive control, BLM cells were first treated with thapsigargin (TG, 1 μM, for 1–24 h), the irreversible SERCA (sarco/endoplasmic reticulum Ca^2+^-ATPase) inhibitor, that disrupts Ca^2+^ homeostasis and activates ER stress^36^; as the experimental model, BLM and A375 cells were treated with δ-TT (20 μg/ml) for 1–24 h. By Western blotting we evaluated the expression levels of ER stress markers (BiP, p-eIF2α, eIF2α, PERK, IRE1α and PDI, protein disulfide isomerase, a Ca^2+^-dependent chaperon protein located in the ER lumen) and ER stress-related apoptosis markers (ATF4, CHOP and ERO1α, a target of CHOP, involved in the hyperoxidation of the ER environment, through generation of ROS). The expression of caspase-4 was also analyzed. Caspase-4 is the initiator caspase involved in the ER stress-induced apoptosis. Following ER stress procaspase-4 is cleaved (e.g. activated) to activate the effector caspase-3.

**Figure 4:**
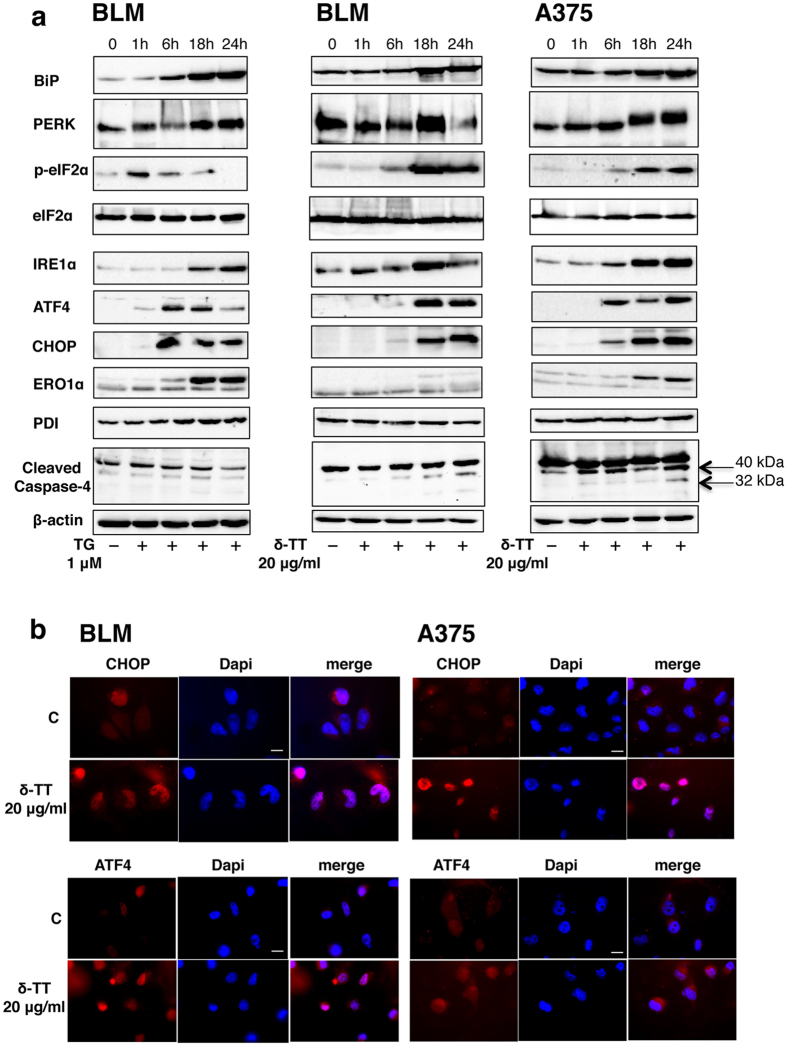
δ-TT increases the expression of ER stress-related proteins in BLM and A375 melanoma cells. (**a**) As a positive control, BLM cells were treated with thapsigargin (TG, 1 μM, for 1–24 h), known to activate ER stress. Melanoma cells (both BLM and A375) were treated with δ-TT (20 μg/ml) for 1–24 h. Western blot analysis was performed with the following antibodies against GRP78/BiP (1:1,500), PERK (1:1,500), p-eIF2α (1:1,000), eIF2α (1:1,000), IRE1α (1:1,000), ATF4 (1:1,000), CHOP (1:1,000), ERO1α (1:1,500), PDI (1:1,000), caspase-4 (1:1,000). β-actin expression was evaluated as a loading control. One representative of three different experiments, for each of the analyses performed, is shown. (**b**) Melanoma cells were treated with δ-TT (20 μg/ml) for 18 h. The intracellular localization of the ER stress-related apoptosis markers ATF4 and CHOP was evaluated by immunofluorescence analysis with the primary antibodies anti-ATF4 (1:100) or anti-CHOP (1:1,000), followed by the TRITC-conjugated secondary antibody and DAPI. C, controls. One representative of three different experiments performed is shown. Scale bar, 5 μm.

As expected, thapsigargin induced a significant, time-dependent, increase in the expression of all these ER stress-related markers in BLM melanoma cells (Fig. 4a). δ-TT induced an increase in the levels of BiP at 18–24 in both BLM and A375 cells. PERK and IRE1α levels were increased at 18 h in BLM cells and at 18–24 h in A375 cells, while p-eIF2α (but not the unphosphorylated form of the protein, eIF2α) levels were increased at 18–24 h of treatment in both cell lines. The levels of the transcription factors ATF4 and CHOP were increased at 18–24 h in BLM cells and at 6–24 h in A375 cells. The expression of ERO1α was unaffected in BLM cells, while its increase was observed in A375 cells. On the other hand, the expression of the chaperone protein PDI was unaffected by the treatment with δ-TT in both cell lines (Fig. 4a). Cleaved caspase-4 levels were also increased by δ-TT treatment (Fig. 4a). This activation occurred at shorter time intervals than caspase-3 cleavage (see Fig. 2b), supporting its role in caspase-3 activation.

To investigate the intracellular localization of the key transcription factors involved in the ER stress process, BLM and A375 cells were treated with δ-TT (20 μg/ml) for 18 h. Figure 4b shows that, in basal conditions, ATF4 and CHOP proteins are expressed at almost undetectable levels in both cells lines. δ-TT treatment induced: 1) the expression of these transcription factors, as evidenced by the appearance of the red fluorescence; 2) their nuclear localization, as shown by the overlapping staining between TRITC-conjugated antibodies and DAPI (Fig. 4b).

### δ-TT increases the expression of ER stress-related mRNAs in melanoma cells

**Figure 5:**
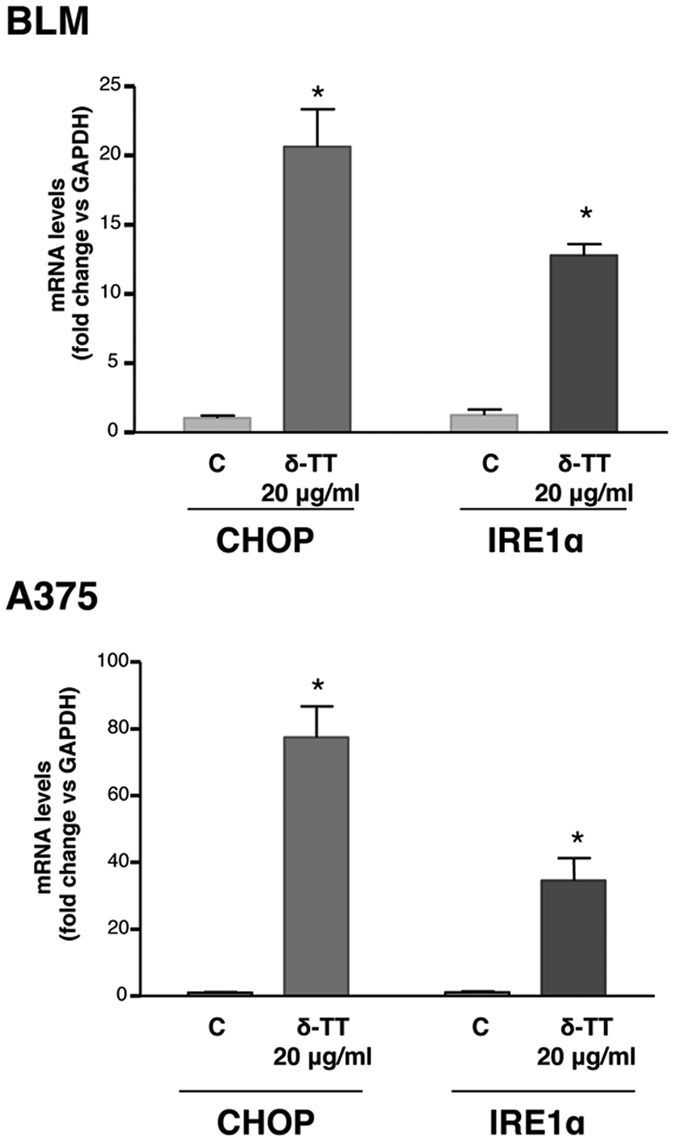
δ-TT increases the expression of ER stress-related mRNAs in BLM and A375 melanoma cells. Melanoma cells were treated with δ-TT (20 μg/ml) for 18 h. The expression levels of CHOP and IRE1α mRNAs were evaluated by quantitiative RT-PCR, utilizing specific sets of primers. Four replicates were performed for each experimental point and the experiments were repeated four times. *p < 0.05 vs C, controls.

To confirm the results obtained from the Western blot analyses, we investigated the effects of δ-TT (20 μg/ml) on the gene expression of CHOP and IRE1α (representative of the two major ER stress-related pathways), measured by real-time RT-PCR. Based on the data on protein expression, the mRNAs levels of the two markers were analyzed after 18 h of treatment with δ-TT. We found that, at this time interval, the mRNA levels of both CHOP and IRE1α were significantly increased, both in BLM and A375 cells (Fig. 5).

### ER stress mediates the δ-TT-induced apoptotic cell death in melanoma cells

**Figure 6:**
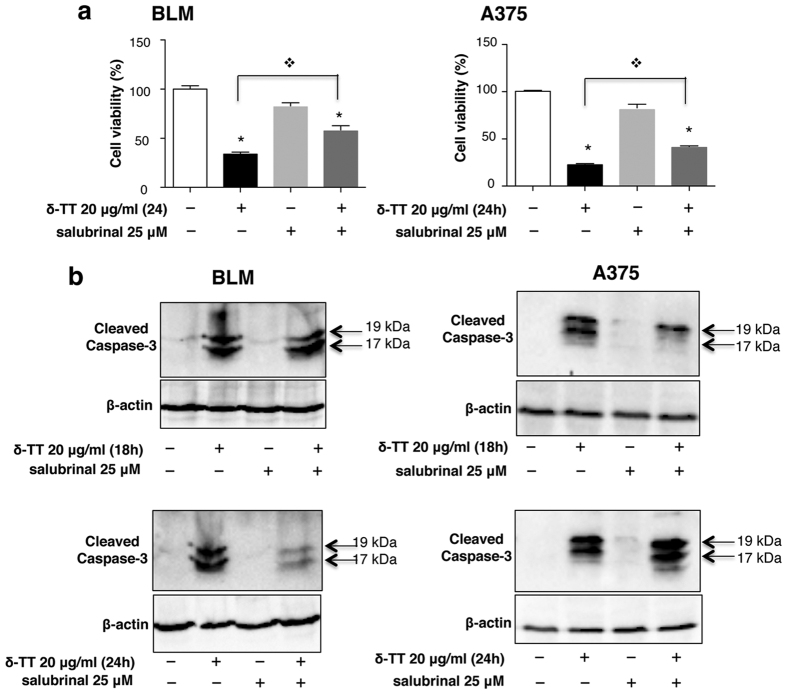
ER stress mediates the δ-TT-induced apoptotic cell death in BLM and A375 melanoma cells. Melanoma cells were pretreated with salubrinal (25 μM), an ER stress-induced apoptosis inhibitor 1 h before δ-TT treatment (20 μg/ml). Cell viability and caspase-3 activation were investigated by means of MTT assay (24 h treatment) (**a**) and Western blot analysis (18 and 24 h treatment) (**b**). In Western blot analysis, β-actin expression was evaluated as a loading control. Each experiment was repeated three times. Data in (**a**) represent mean values ± SEM and were analyzed by Bonferroni’s test after one-way analysis of variance. *p < 0.005 vs controls. 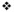
p < 0.005 vs δ-TT.

Data so far reported suggest that ER stress might be involved in the proapoptotic activity of δ-TT. To confirm this hypothesis, BLM and A375 cells were pretreated with 25 μM salubrinal (an ER stress-induced apoptosis inhibitor, due to its inhibitory activity on eIF2α dephosphorylation)^37^, 1 h before δ-TT. Cell viability and caspase-3 activation were then investigated by means of MTT assay and Western blotting, respectively. Figure 6a shows that, as expected, cell viability was significantly decreased after δ-TT treatment; salubrinal alone did not affect cell viability (24 h). Pretreatment with salubrinal significantly reverted the cytotoxic effect of δ-TT, both in BLM and in A375 cells. These results were confirmed by caspase-3 cleavage (at 24 and 18 h in BLM and A375 cells, respectively) (Fig. 6b). It must be underlined that the cytotoxic effect of δ-TT was significantly, but not completely, reverted by salubrinal. This suggests that different apoptotic mechanisms, in addition to the PERK/p-eIF2α/ATF4/CHOP pathway, might be involved in the antitumor activity of δ-TT, particularly in A375 cells at this time interval (24 h). In support to this hypothesis, Fig. 6b shows that the expression levels of cleaved caspase-3 are still high in this cell line at 24 h of treatment.

### Growth-inhibitory activity of δ-TT on melanoma xenografts in nude mice

**Figure 7:**
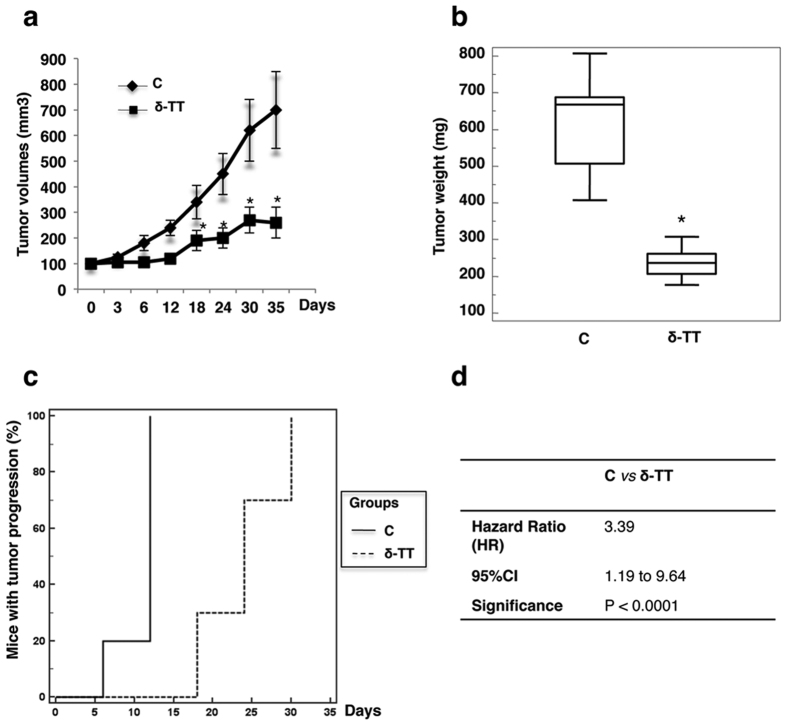
δ-TT inhibits the growth and progression of A375 melanoma xenografts in nude mice. Mice xenografted with A375 cells were treated orally with δ-TT (100 mg/kg in olive oil, daily, 5 days/week) for different time intervals, up to 35 days, starting when the tumor volume reached 0.5–0.8 mm^3^. (**a**) Tumor volume was assessed at the beginning of the treatment, at 3, 6, 12, 18, 24, 30, and 35 days, the end of the treatment. *p < 0.001 vs C, controls. (**b**) Tumor weight was assessed at the end of the treatment. *p < 0.0001 vs C, controls. (**c**) Graphical representation of tumor progression incidence, determined by Kaplan-Meier analysis, over the time upon δ-TT treatment. (**d**) The curves reported in (**c**) were compared by the Logrank test to evaluate the respective hazard ratio values, 95% confidence in hazard ratio and significance values. C, controls.

Based on the significant antitumor activity of δ-TT on melanoma cells *in vitro*, preclinical experiments were performed on animal models. Since δ-TT exerted a similar proapoptotic activity on both melanoma cell lines (e.g. independently on their mutation status), we selected the A375 cell line to perform these experiments. A375 cells were subcutaneously inoculated in 6 months-old female immunodeficient CD1-nu/nu mice. At 10 days after tumor injection, mice were orally treated with δ-tocotrienol extract in olive oil (100 mg/kg daily, 5 days/week) up to 35 days, while controls received olive oil only. Tumor volumes were calculated at the beginning of the treatment, at 3, 6, 12, 18, 24, 30, and 35 days, the end of the treatment: a significant reduction was observed at 24–35 days, comparing the δ-TT-treated group with the control group (Fig. 7a). At the end of the experiments (day 35) a significant decrease (60.6%) of tumor weight was observed in δ-TT-treated *vs*. control mice, as evaluated by Mann-Whitney test (Fig. 7b). The incidence of tumor progression was also determined by Kaplan-Meier analysis, evidencing a delay of tumor progression in δ-TT-treated group (Fig. 7c). In control mice tumor progression occurred 6 days after treatment with vehicle and progressively increased to 100% on day 12 (Fig. 7c). In contrast, in δ-TT-treated mice, tumor progression occurred at day 18 and progressively increased to 100% on day 30 (Fig. 7c). The mean time to tumor progression was significantly delayed in δ-TT-treated mice (24 days; 95%CI 9.23 to 12.37) with respect to controls (10.8 days; 95%CI 20.96 to 27.04). The curve reporting the incidence of tumor progression over time during δ-TT treatment significantly differed from controls (Logrank test; P < 0.0001). Mice treated with vehicle developed 3.30 fold-higher probability to develop tumor progression than mice treated with δ-TT (HR = 3.39; 95%CI 1.19 to 9.64; P < 0.0001, Fig. 7d).

## Discussion

Vitamin E-derived TTs exert healthy effects in various chronic pathologies, such as cardiovascular and neurodegenerative diseases, based on their antiinflammatory and antioxidant properties. TTs, the δ-TT and γ-TT isomers in particular, were also shown to exert anticancer activity in different tumor models. On the other hand, evidence so far available about the antitumor effects of TTs on melanoma, and the molecular mechanisms underlying this activity, are still scanty.

We sought to investigate whether δ-TT might exert a cytotoxic/proapoptotic effect in two human melanoma cells lines (BLM and A375) and to dissect the molecular mechanisms underlying this activity. The data obtained from *in vitro* studies were then translated into *in vivo* experiments. For these studies we selected BLM and A375 human melanoma cell lines, representative of the most frequent genetic mutations in melanomas (*NRAS* and V600E *BRAF* in BLM and A375 cells, respectively).

We found that δ-TT, significantly and dose-dependently, reduces cell viability in both melanoma cell lines; importantly, the tocotrienol did not exert toxic effects on normal melanocytes at any of the doses utilized, suggesting that it may act by selectively targeting cancerous cells. We confirmed the δ-TT cytotoxic activity on both BLM and A375 cells by the reduction in the viability of colony-forming cells and the induction of cell death by the Trypan blue-exclusion assay. We further demonstrated that δ-TT triggers the intrinsic apoptosis pathway as evidenced by the increased Bax/Bcl-2 ratio, cytochrome *c* release from mitochondria and cleavage of procaspase-3 and its downstream target PARP in both cell lines.

These results demonstrate that δ-TT exerts a significant antiproliferative/proapoptotic effect on human melanoma cells; importantly the tocotrienol is devoid of any cytotoxic activity on normal melanocytes.

In line with our observations, δ-TT was reported to induce cell cycle arrest and apoptosis in human melanoma cells^38^,^39^, while γ-TT was shown to induce apoptosis, suppress invasion and sensitize cells to chemotherapeutic drugs^40^. Finally, the antitumor activity of TTs was observed in B16 murine melanoma cells^41^,^42^,^43^.

The observation that δ-TT is associated with a significant proapoptotic effect in melanoma cells is in agreement with the reported antitumor activity of TTs (mainly δ-TT and γ-TT) in different types of tumors^19^,^33^,^35^,^44^.

To get insights into the molecular mechanisms involved in the proapoptotic activity of TTs in melanoma cells, we focused our attention on the ER stress pathways. As outlined in the Introduction, in conditions of severe ER stress, the chaperone protein BiP dissociates from the ER membrane and triggers the activation of the three main protein sensors (PERK, IRE1α and ATF6); each of these proteins is coupled with a cytosolic pathway converging on apoptosis^26^,^28^. We found that, in both BLM and A375 melanoma cell lines, δ-TT increases the expression of BiP and of the two sensors PERK and IRE1α. In line with this observation, δ-TT also increased the expression levels of p-eIF2α and the expression/activation (cytoplasmic-to-nuclear localization) of the transcription factors ATF4 and CHOP. p-eIF2α, ATF4 and CHOP are known to be induced by PERK; in addition, CHOP was shown to be activated by IRE1α through the p38MAPK pathway^26^,^28^. In agreement with these observations we found that the expression of CHOP and IRE1α was increased by δ-TT also at the mRNA level. Taken together, these data indicate that both the PERK and IRE1α signaling branches are triggered in melanoma cells after δ-TT treatment, converging to the activation of CHOP, the key mediator of the ER stress-related apoptosis.

We observed that δ-TT increases the expression of ERO1α in A375, but not in BLM, melanoma cells; on the other hand the expression of PDI was unaffected by the treatment in both cell lines. ERO1α, a direct target of CHOP, is involved in the hyperoxidation of the ER environment through generation of ROS and in the protein folding processes through activation of PDI. To explain our results, we suggest that, in A375 cells, the increase of ERO1α levels might reflect an overproduction of ROS, contributing to cell stress^45^. On the other hand, the lack of ERO1α induction in BLM cells might indicate a reduced activity of PDI in restoring protein folding, leading to an ER stressful condition that cannot be attenuated.

Caspase-4 is the initiator caspase directly involved in the ER stress-induced apoptosis^28^ through the activation of the effector caspase-3. Different ER stressors lead to the release of ER Ca^2+^ stores, leading to the activation of calpain^28^ that, in turn, cleaves caspase-4; caspase-4 can also be activated by IRE1α in conditions of severe ER stress. Here, we found that δ-TT induced the cleavage of caspase-4 in both BLM and A375 melanoma cell lines.

These data demonstrate that, in melanoma cells, δ-TT triggers cell death and activates the ER stress-related pathways. ER stress-related apoptosis markers, particularly the PERK/p-eIF2α/ATF4/CHOP and IRE1α branches, were previously demonstrated to induce cell death through the activation of the intrinsic apoptosis pathway^26^,^28^. To verify whether the proapoptotic activity of δ-TT might be mediated by the ER stress process, we evaluated the effects of salubrinal on the activity of the tocotrienol on melanoma cell viability and on caspase-3 activation. Salubrinal is an inhibitor of the ER stress-related apoptosis pathways, due to its inhibitory effect on the eIF2α dephosphorylation^37^. Thus, salubrinal is considered a legitimate candidate drug to counteract the effects of cytotoxic compounds that induce ER stress. Our data demonstrate that, in BLM and A375 cells, salubrinal significantly counteracts the cytotoxic activity of δ-TT as evaluated in terms of cell viability and caspase-3 activation. However, in both cell lines, the reversal of the apoptotic activity of δ-TT was only partial. This may be explained, at least partially, by the fact that salubrinal targets only the PERK branch of the ER stress.

Based on the *in vitro* results, we sought to confirm the efficacy of δ-TT against melanoma growth in preclinical models of A375 melanoma cell xenografts in nude mice. We observed that δ-TT induces a significant reduction in tumor volume at different times of treatment as well as in tumor mass at the end of the treatments; importantly, δ-TT also significantly delayed tumor progression. No systemic toxicity was observed, supporting the above discussed lack of cytotoxicity of δ-TT on normal human melanocytes.

The data here reported demonstrate that δ-TT exerts a significant proapoptotic activity on human melanoma cells (while sparing normal melanocytes), both *in vitro* and *in vivo*, and that this activity is mediated, at least partially, by the induction of the ER stress process. To our knowledge, the association of the ER stress with the apoptotic activity of tocotrienols has been demonstrated only in breast cancer cells, so far^46^,^47^. On the other hand, the ER stress branches have been shown to be involved in the antitumor activity of a variety of natural compounds^48^,^49^,^50^,^51^.

ER stress-mediated pathways were proved to represent a relevant target for melanoma treatment^52^; moreover, drugs that specifically act by targeting the ER stress were shown to improve the efficacy of chemotherapeutic drugs^53^. Finally, the biosafety of TTs was previously reported^12^. Moreover, clinical trials are at present ongoing with the aim to evaluate the safety, biodisponibility and efficacy of TTs in different tumors (pancreatic, lung and ovarian cancers), given either alone or in association with standard treatments (ClinicalTrials.gov). Based on these observations, our results support the evidence that δ-TT, through its ability to trigger the ER stress, might be considered as an effective compound for novel chemopreventive/therapeutic (e.g. combinational) strategies for melanoma.

## Methods

### Cell cultures

The human BLM melanoma cell line (*NRAS* mutated) was provided by Dr. G.N. van Muijen (Department of Pathology, Radbound University Nijmegen Medical Center, Nijmegen, The Netherlands). This cell line is a subline of BRO melanoma cells isolated from lung metastases after subcutaneous inoculation of nude mice with BRO cells^54^. This cell line was previously utilized in the authors’ laboratory^55^. The human A375 melanoma cell line (V600E *BRAF* mutated) was from American Type Culture Collection (ATCC, Manassas, VA, USA). Human primary melanocytes were provided by Dr. F. Crovato (Regional Reference Centre for Human Epidermis *in vitro* Culture and Bank for Tissue Crypreservation, Niguarda Hospital, Milano, Italy). Original stocks of cells were stored frozen in liquid nitrogen; after resuscitation, cells were kept in culture for no more than 10–12 weeks. BLM and A375 melanoma cells, and human melanocytes were routinely cultured in DMEM medium supplemented with 10% FBS, glutamine and antibiotics. Cells were cultured in humidified atmosphere of 5% CO_2_/95% air at 37 °C.

### Materials

For Western blotting analysis the following primary antibodies were utilized: cleaved caspase-3 (Asp175; clone 5A1E), PARP, BiP (clone C50B12), PERK (clone D11A8), p-eIF2α (clone D9G8), eIF2α (clone D7D3), IRE1α (clone 14C10), ATF4 (clone D4B8), CHOP (clone L63F7), ERO1α, PDI (clone C81H6) and caspase-4 (Cell Signaling Technology Inc., Boston, MA); Bcl-2 (clone C-2), Bax (clone 6A7) and cytochrome *c* (clone 7H8) (Santa Cruz Biotecnology Inc., Santa Cruz, CA). Horseradish-peroxidase-conjugated secondary antibody and enhanced chemiluminescence reagents were from GE Healthcare, Life Sciences (Milano, Italy). For immunofluorescence analysis the following primary antibodies were utilized: ATF4 (clone D4B8), CHOP (clone L63F7) and cytochrome *c* (clone 7H8) (Santa Cruz Biotechnology Inc.). FITC- or TRITC-conjugated secondary antibodies Alexa Fluor 488 and 594 were from Molecular Probes Inc. (Eugene, OR). Salubrinal (the selective eIF2α dephosphorylation inhibitor), thapsigargin (the SERCA inhibitor), and all analytical grade solvents were from Sigma-Aldrich (Milano, Italy).

### δ-TT purification

HPLC analysis and isolation of δ-TT were done using a LC-940 Liquid Cromatography instrument (Varian, Leinì, Italy) equipped with: binary pump system (pump head volume V = 10 ml, eluent flow range: 0.3–25 ml/min), autosampler (5 ml vials, injection volume 5–4000 ml), scale up module, photodiode array (wavelengths range: λ = 200–400 nm, set at λ = 290 nm), automatic fraction collector. Separations were conducted using an analytical reversed phase Kinetex^®^ column (5 μm C18 100 Å, 100 × 4.6 mm, Phenomenex, Castel Maggiore, Italy), eluent flow 2 ml/min (injection volume 5 μl). For the isolation of δ-TT at semipreparative scale, a Kinetex AXIA column (5 μm C18 100Å, 100 × 21.5 mm, Phenomenex, Castel Maggiore, Italy) was used. Eluent: acetonitrile 100%, flow: 20 ml/min, run time: 10 min, injection volume: 4000 μl. δ-TT was isolated from: a) a commercial palm oil (*Elaeis guineensis*) fraction enriched in tocotrienols/tocopherols (Gold Tri E 70% w/w, Golden Hope Bioganic, Selangor, Malaysia), for *in vitro* studies; b) a commercial extract of Annatto (*Bixa orellana*) seeds (DeltaGold, American River Nutrition Inc., Hadley, MA), for *in vivo* studies. a) The palm oil fraction was extracted with acetonitrile (1:1 vol/vol), the solvent phase centrifuged (6000 rpm, d = 15 cm, t = 5 min) and evaporated under reduced pressure (endpoint 1 mbar) at room temperature. The oily extract (100 mg, OE) was dissolved in HPLC grade methanol (5 ml) injected as above described. Fractions were collected in time slice mode in the RT window 2.3–2.5 min. To eliminate the contamination from the partially overlapping peak due to β- and γ-TT, all fractions of interest were pooled together, acetonitrile evaporated, the resulting residue diluted again in methanol and reinjected until a purity of at least >95% was achieved. The average recovery yield was around 10 mg per 100 mg of OE. Aliquots of 50 mg were diluted in dimethylsulfoxide at the concentration of 50 mg/ml and stored at −20 °C. b) Annatto extract was processed as in (a) with minor modifications. deltaGold (200 mg) was extracted with methanol (5 ml), centrifuged and the supernatant injected and processed as in (a). Aliquots of purified δ-TT were pooled together and diluted in olive oil.

### MTT viability assay

BLM and A375 melanoma cells were seeded at a density of 3 × 10^4^ cells/well and human melanocytes at a density of 10^4^ cells/well in 24-well plates. After 24 h, cells were treated with different doses of δ-TT (5–20 μg/ml) for 24 or 48 h. The medium was changed with MTT solution (0.5 mg/ml) in DMEM without phenol red and FBS; cells were incubated at 37 °C for 15–45 min and violet precipitate was dissolved with isopropanol. Absorbance at 550 nm was measured through an EnSpire Multimode Plate reader (PerkinElmer, Milano, Italy).

To assess the ER-stress involvement in the δ-TT-induced cell death, BLM and A375 cells were seeded at a density of 3 × 10^4^ cells/well in 24-well plates, in DMEM complete medium. After 48 h, cells were treated with salubrinal (25 μM). After 1 h treatment, 20 μg/ml of δ-TT or vehicle were added to each well. After 24 h, cell viability was analyzed as describe above.

### Trypan blue exclusion assay

BLM and A375 cells were plated (5 × 10^4^ cells/dish) in 6-cm dishes. After 48 h cells were treated with different doses of δ-TT (5, 10, 15 or 20 μg/ml) for 24 h. Adherent and floating cells were then harvested, stained with Trypan blue 0.4% (1:1 v/v) and counted by Luna automated cell counter (Logos Biosystems, Annandale, VA), discriminating between total, viable and dead cells.

### Colony-formation assay

BLM and A375 cells were seeded (100–250 cells/well, depending on the cell type) in 6-well plates. After 48 h, cells were treated with δ-TT (20 μg/ml) for 72 h and then cultured for 7–10 days. Colonies were fixed with 70% methanol and stained with Crystal Violet 0.15%. Images of stained colonies were captured by a Nikon photocamera.

### Western blot assay

To investigate the effects of δ-TT on the expression of apoptosis and ER stress-related proteins, BLM and A375 cells were plated (5 × 10^5^ cells/dish) in 10-cm dishes, for 48 h. Cells were then treated with δ-TT (20 μg/ml) for 1–48 h. Adherent and floating cells were harvested and lysed in RIPA buffer; protein preparations (30–40 μg) were resuspended in reducing Sample buffer (Bio-Rad Laboratories, Segrate, Mi, Italy) and heated at 95 °C for 5 min. Following electrophoretic separation by SDS-PAGE, proteins were transferred onto nitrocellulose membrane. After blocking, membranes were incubated with the primary antibodies against cleaved (activated) caspase-3, PARP, Bcl-2, Bax, BiP, PERK, p-eIF2α, eIF2α, IRE1α, ATF4, CHOP, ERO1α, PDI, caspase-4. Detection was done using horseradish peroxidase-conjugated secondary antibodies, and enhanced chemiluminescence ECL-Prime reagents. As a positive control of ER stress induction, BLM cells were treated with 1 μM thapsigargin, a selective inhibitor of the sarco/endoplasmic reticulum Ca^2+^-ATPase (SERCA). The expression of ER stress-related proteins was analyzed as above.

To assess the ER-stress involvement in the δ-TT-induced apoptosis, BLM and A375 cells were seeded at a density of 5 × 10^5^ cells/dish. After 48 h, cells were treated with salubrinal (25 μM). After 1 h of treatment, 20 μg/ml of δ-TT were added to each well. After 24 h, the expression levels of cleaved caspase-3 were assessed by Western blot assay as described above.

### Immunofluorescence assay

To assess the localization of ER stress markers, BLM and A375 cells were seeded at 3 × 10^4^ cells/well on polylysine-coated 13-mm diameter coverslips. After 48 h, cells were treated with δ-TT (20 μg/ml) for 18 h, fixed and stained with ATF4 or CHOP antibodies, followed by TRITC-conjugated secondary antibody and DAPI.

To verify the release of cytochrome *c* from mitochondria, BLM and A375 cells were seeded as described, followed by 24 h treatment with δ-TT. Cells were then incubated for 30 min with Mitotracker (250 nM) (Molecular Probes Inc.), fixed and stained with cytochrome *c* antibody, followed by FITC-conjugated secondary antibody and DAPI. Labeled cells were examined under a Zeiss Axiovert 200 microscope with a 63x/1.4 objective lens linked to a Coolsnap Es CCD camera (Roper Scientific-Crisel Instruments, Roma, Italy).

### Quantitative PCR

To investigate the effects of δ-TT on the expression of ER stress-related (CHOP and IRE1α) mRNAs, BLM and A375 cells were plated (1.5 × 10^5^ cells/well) in 6-well plates, for 48 h. Cells were then treated with δ-TT (20 μg/ml) for 18 h. Adherent and floating cells were harvested and total RNA was extracted using TRIzol according to the manufacturer’s instructions (Invitrogen Life Technology, Inc., Paisley, UK). RNA pellet concentrations were assessed spectrophotometrically using Nanodrop 2000 (Thermo Fisher Scientific, Waltham, MA) (OD 260/280).

Specific sets of primers for CHOP and IRE1α cDNAs were designed and synthesized (Sigma-Aldrich), according to Chen and coworkers^56^. Real-time PCR was performed as previously described^57^. Briefly, real-time DNA amplification was performed in CFX96 Bio-Rad using 20 μl of total volume. The efficiency of each set of primers was evaluated in preliminary experiments and it was found close to 100% for target genes and for the housekeeping gene glyceraldehyde-3-phosphate dehydrogenase (GAPDH). Total RNA (600 ng) was retrotranscribed using the IScript Supermix kit (Bio-Rad), according to the manufacturer’s protocol. The amplification was carried out on 2.5 ng of total cDNA using SYBR chemistry (iTAQ Universal SYBR green supermix, Bio-Rad) according to the manufacturer’s protocol. Real-time PCR was run according to the following protocol: an initial step of 30 sec at 95 °C followed by 40 cycles of 5 sec at 95 °C and 30 sec at 60 °C. A dissociation stage with a melt curve analysis was also performed. Four replicates were performed for each experimental point and experiments were repeated four times. Gene expression was quantified using the comparative threshold-cycle (DDCt) method considering that the targets and the reference genes have the same amplification efficiency (near to 100%).

### Mouse melanoma xenografts

Immunodeficient female CD1-nu/nu mice, at 6 weeks of age, were purchased from Charles River (Milano, Italy). All the experiments were approved and were carried out in accordance with the relevant guidelines and regulations established by the University of L’Aquila (Medical School and Science and Technology School Board Regulations, complying with the Italian government regulation n.116 January 27 1992 for the use of laboratory animals) which is in line with ARRIVE guidelines^58^. Before manipulations, all mice were anesthetized with a mixture of ketamine (25 mg/ml) and xylazine (5 mg/ml) and received subcutaneous flank injections (2 each) of 10^6^ A375 cells mixed with Matrigel (Beckton Dickinson Labware, Bedford, MA, USA). At 10 days after tumor injection, mice with tumor volume 0.5–0.8 cm^3^ were divided into two groups receiving olive oil (10 tumors) or δ-TT (100 mg/kg in olive oil, daily; 10 tumors) by oral gavage, 5 days/week. At the end of the experiments (35 days after the start of treatments) animals were sacrificed by carbon dioxide inhalation and tumors were surgically removed. Tumor growth was assessed at the beginning of the treatment, at 3, 6, 12, 18, 24, 30 and at 35 days by measuring tumor diameters with a Vernier caliper (length × width). The volume of the tumor was expressed in mm^3^ according to the formula 4/3 πr^3^. Tumor weight was calculated according to the formula: TW (mg) = (d^2^ × D)/2, where d and D are the shortest and longest diameters, respectively^59^. Tumor progression (TP) was defined as an increase of tumor volume greater than 100% with respect to baseline; time to tumor progression (TTP). To evaluate treatment toxicity, serial bodyweight measurements were performed every 3–4 days during treatments.

### Statistical analysis

When appropriate, data were analyzed by Bonferroni’s test, after one-way analysis of variance. The incidence of tumor progression was determined by Kaplan–Meier analysis and a Gehan’s generalized Wilcoxon test. Two Kaplan–Meier curves were compared by the Logrank test. All tests were two-sided and were determined by Monte Carlo significance. P values < 0.05 were considered statistically significant. SPSS (statistical analysis software package, IBM Corp., Armonk, NY, USA) version 10.0 and StatDirect (version. 2.3.3, StatDirect Ltd, Altrincham, Manchester, UK) were used for statistical analysis and graphical presentation.

## Additional Information

**How to cite this article**: Montagnani Marelli, M. *et al*. Vitamin E δ-tocotrienol triggers endoplasmic reticulum stress-mediated apoptosis in human melanoma cells. *Sci. Rep*. **6**, 30502; doi: 10.1038/srep30502 (2016).
